# Geographic disparities and temporal trends in aggressive prostate cancer: a SEER population-based analysis (2004–2022)

**DOI:** 10.1007/s10552-026-02267-5

**Published:** 2026-10-17

**Authors:** Yifei Xu, Ayush Singh, Eric Li, Nicholas George Zaorsky, Nathaniel Geyer, Yifan Yang, Yanxu Yang, Alicia C. McDonald, Lijun Zhang, Ming Wang

**Affiliations:** 1 Department of Population and Quantitative Health Sciences, Case Western Reserve University School of Medicine, 10900 Euclid Avenue, Cleveland, OH 44106 USA; 2 Department of Radiation Oncology, University Hospitals Seidman Cancer Center, Case Western Reserve University School of Medicine, 11100 Euclid Ave, Cleveland, OH USA; 3 The University of Texas at Austin, 2515 Speedway, Austin, TX 2515 USA; 4 Department of Radiation Oncology, Mayo Clinic Florida, 4500 San Pablo Road, Jacksonville, FL 32224 USA; 5 Department of Public Health Sciences, Pennsylvania State University College of Medicine, 500 University Drive, Hershey, PA 17033 USA; 6 Department of Pediatrics, Emory University School of Medicine, 2015 Uppergate Drive, Atlanta, GA 30322 USA

**Keywords:** Prostate cancer aggressiveness, Metastasis, Age-adjusted incidence, Rurality, Appalachia

## Abstract

**Purpose:**

Prostate cancer burden may be amplified in rural regions or Appalachia areas, where access to screening and healthcare may be limited. This study examined geographic disparities and temporal trends in aggressive and metastatic prostate cancer across four groups defined by rurality and Appalachian status, while assessing assessed racial differences.

**Methods:**

We conducted a retrospective study using the Surveillance, Epidemiology, and End Results (SEER) including newly diagnosed prostate cancer men aged ≥40 years (2004–2022). Age-adjusted incidences of more aggressive and metastatic cases were estimated for each geographical region. Multivariable logistic regression was used to evaluate geographic and racial disparities in risk of more aggressive cases, adjusting for demographic and clinical factors.

**Results:**

We identified 951,965 eligible cases. Overall prostate cancer incidence declined in most regions, with a decrease in rural Appalachia (Annual percent changes [APC] = − 1.7%; 95% CI: [− 2.98%, − 0.42%]). Incidence of more aggressive cases increased over time particularly in urban Appalachia (APC = 2.73%; 95% CI: [1.20%, 4.65%]). For metastatic cases, urban Appalachia had the lowest overall incidence and rural non-Appalachia the highest, and rates increased across regions after 2020. In multivariable regression, geographic patterns differed by race. Among White men, compared with urban non-Appalachia, the odds of more aggressive prostate cancer were higher in rural non-Appalachia and rural Appalachia but lower in urban Appalachia. However, among Black men, those residing in rural Appalachia had significantly lower odds than those residing in urban non-Appalachia. Older age, Hispanic ethnicity, higher serum PSA, and lymph node-positive status were also associated with higher odds of more aggressive prostate cancer.

**Conclusion:**

Prostate cancer burden and severity varied by Appalachian status and rurality, supporting targeted, region- and race-specific strategies to improve early detection and access to high-quality care.

**Supplementary Information:**

The online version contains supplementary material available at https://doi.org/10.1007/s10552-026-02267-5.

## Introduction

Prostate cancer is the second most commonly diagnosed cancer and a leading cause of cancer-related death among men in the United States [1, 2]. Although many prostate cancers have an indolent, initially asymptomatic course, prostate cancer remains a major public health concern [2]. The American Cancer Society estimated approximately 299,010 new prostate cancer cases and 35,250 prostate cancer deaths in the United States in 2024 [3]. These figures are particularly alarming given the disproportionately higher incidence and greater likelihood of aggressive disease observed in medically underserved populations (e.g., men in rural areas with limited access to care) and in high-risk groups (e.g., Black men and men with elevated inherited/genetic risk) [4–7]. Socioeconomic and structural factors such as geographic location, race, income, education, and access to healthcare resources may contribute to disparities in prostate cancer development, diagnosis, and progression [8–10]. In particular, geographic context has been linked to differences in cancer incidence and survival [11–13]. Individuals in rural areas may face barriers to timely cancer screening, leading to later-stage diagnoses and poorer prognoses compared with urban populations [13, 14]. In addition, disparities in healthcare infrastructure and limited availability of specialized cancer care, driven in part by shortages of urologists, may contribute to regional differences in outcomes [15]. These geographic variations highlight the importance of tailored public health strategies and resource allocation to improve cancer outcomes.

The Appalachian region, which spans 13 states along the Appalachian Mountains [16, 17], often have poorer health outcomes including cancer [11, 12]. However, findings on prostate cancer health outcomes (e.g., incidence, aggressiveness, and mortality) in this region have been inconsistent [18–20]. For example, in Appalachian Kentucky, prostate cancer incidence was reported to be lower than in non-Appalachian Kentucky (113 vs. 137 per 100,000), yet mortality was higher (23.8% vs. 21.8%). Appalachian counties also had higher PSA and Gleason scores, more advanced disease, greater comorbidity burden, lower education, and higher poverty, suggesting that the observed incidence-mortality pattern may be driven more by socioeconomic conditions, comorbidities, and access to timely, high-quality care than by geography alone [21]. Conversely, one of our prior studies comparing Appalachian and non-Appalachian regions in Pennsylvania reported lower prostate cancer incidence and a lower prevalence of aggressive disease in Appalachian areas, potentially related to lower screening uptake in Appalachia. For example, the Appalachian Community Cancer Network reported that among Pennsylvania men aged ≥50 years, those living in Appalachian counties were 1–2% points less likely to have ever received a serum PSA test or digital rectal examination than age-matched men residing in non-Appalachian counties [19, 22]. Of note, we consider cancer aggressiveness using a composite of measures assessed at diagnosis, including Gleason score, tumor stage, and distant metastasis (see details below), aiming to capture clinically meaningful disease severity. These studies highlight important disparities in Appalachian population, yet it remains unclear how living in Appalachian affects prostate cancer incidence and aggressiveness. National data may provide further insight into these inequities [23–27].

In this study, we investigated the incidence of prostate cancer and its aggressiveness across four geographic groups defined by rurality and Appalachian status and evaluated temporal trends and racial disparities. We hypothesized that the odds of more aggressive prostate cancer differ across these geographic groups and that disparity patterns vary by race. Specifically, men residing in rural areas, particularly within Appalachian regions, would have higher odds of being diagnosed with more aggressive prostate cancer than those living in urban areas.

## Materials and methods

### Study population

We conducted a cross-sectional study of men age 40 years or older diagnosed with primary prostate cancer from 2004 to 2022, using data from the Surveillance, Epidemiology, and End Results (SEER) database (RRID: SCR_006902). Specifically, we used the SEER Research Plus Specialized Data (Nov 2024 submission), which includes cancer diagnoses from 2000 to 2022 across SEER 17 registries (excluding Alaska) and covers approximately 26.5% of the U.S. population with expanded race/ethnicity variables. A key advantage of this specialized dataset is the inclusion of county-level Federal Information Processing Standards (FIPS) codes in the case-listing output, enabling linkage to external county-level attribute data (e.g., demographic and socioeconomic indicators) for contextual and disparities analyses.

**Fig. 1:**
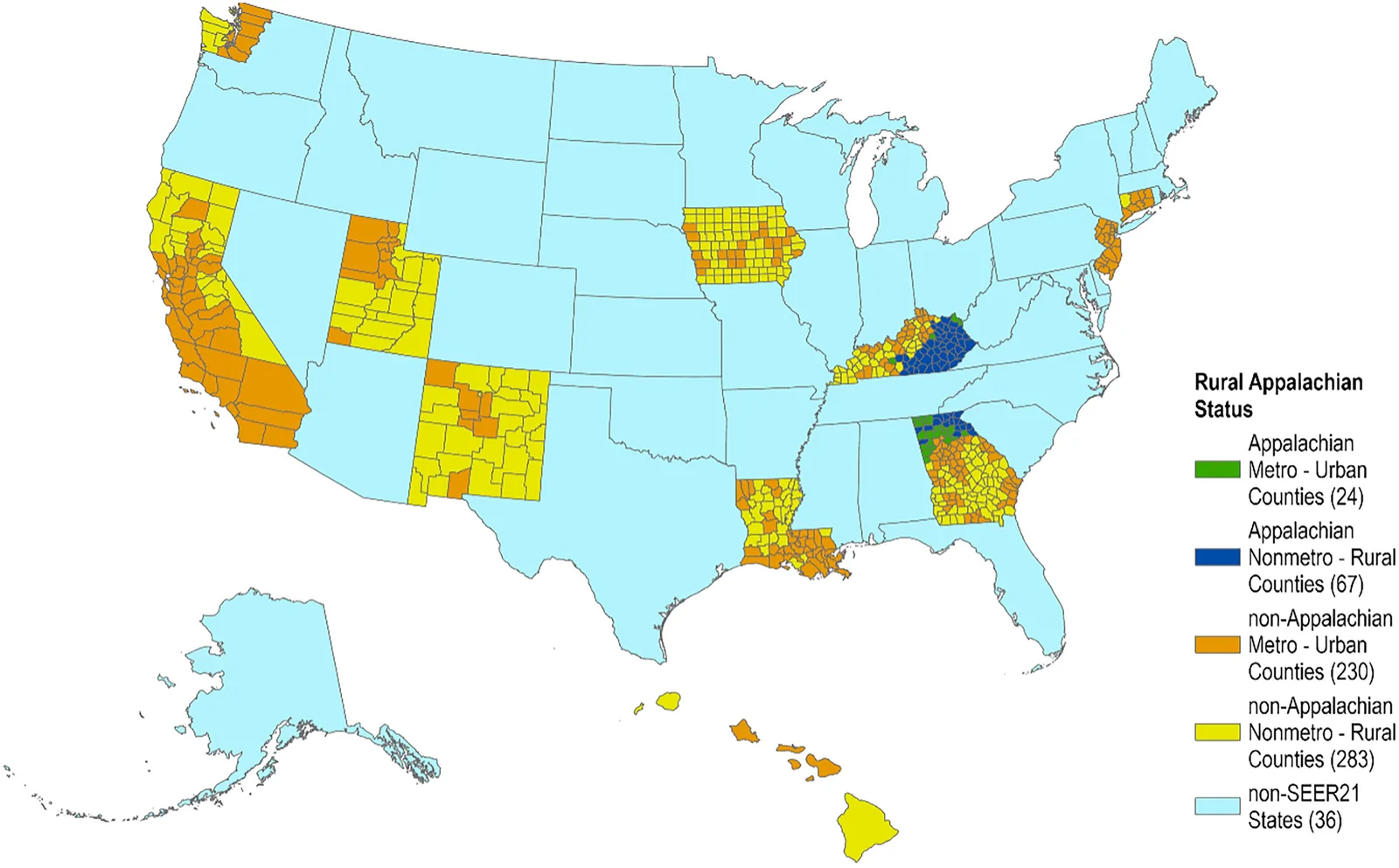
The GIS map of counties by Appalachia and rurality for SEER 2004–2022

For our analysis, we conducted data cleaning and processing to extract factors of interest, with a particular focus on the geographic disparity study. Appalachian regions were defined according to the Appalachian Regional Commission’s 2008 definition (RRID: SCR_027475). Also, we used the US Department of Agriculture’s 2013 Urban Rural Continuum Codes RUCC; RRID: SCR_011486) to classify Appalachian and non-Appalachian regions into metro (urban) areas, coded 1–3, and non-metro (rural) areas, coded 4–9, based on population size, degree of urbanization, and proximity to metropolitan areas [28, 29] (Fig. 1). Other study demographic and clinical variables included age at diagnosis, serum prostate-specific antigen (PSA) levels at diagnosis based on laboratory values, race (White, Black, Others), ethnicity (Hispanic, Non-Hispanic), lymph node positive status (Yes, No), marital status (Ever Married or Living as Married, Single—Never Married), Gleason score, tumor stage (T1, T2, T3, T4), presence of metastasis (Yes, No), and definitive treatment received (Yes if the patient received surgery, radiation, or hormone therapy; otherwise, No). Also, distant metastasis was defined according to the American Joint Committee on Cancer (AJCC, 7th Edition) staging, incorporating the Cervical and Supraclavicular (CS) lymph nodes as well as the AJCC pathologic staging based on physical examination, imaging studies, and/or other non-invasive clinical evidence. To minimize missing data, we derived key disease characteristics using all available related SEER variables and supplemental fields. For tumor stage, metastasis, and Gleason score, information from multiple sources was combined. For example, tumor stage was primarily based on pathologic stage, with clinical stage used when pathologic stage was unavailable. More aggressive prostate cancer was defined using a composite of measures assessed at diagnosis, including Gleason score 7 (4+3) or 8–10, T3-T4 tumor stage, or distant metastasis. If at least one of these three criteria was known to be met, though some variables were missing, the case was classified as more aggressive. For categorical covariates with missing values, an “Unknown” category was created to retain these observations in the analysis.

Our primary outcome is prostate cancer aggressiveness, which was determined based on clinical or pathological Gleason score, tumor stage, and distant metastasis at the time of diagnosis, similarly to our previous work [7, 19, 22, 30]. Men with a Gleason score <6 were excluded. Less aggressive cancer was defined as cases with a Gleason score of 6 or 7 (3+4 pattern) and tumor stage T1-T2 with no distant metastasis. More aggressive cancer was defined as cases with a Gleason score of 7 (4+3 pattern), 8, 9, or 10, or tumor stage T3-T4 or having distant metastasis. The secondary outcome will be distant metastasis only at diagnosis.

### Data analysis

Descriptive statistics for demographic and clinical characteristics were summarized overall and by geographical region defined by rurality and Appalachian status (Urban Appalachian, Rural Appalachian, Urban Non-Appalachian, Rural Non-Appalachian) as means and standard deviations for continuous variables, and frequencies with percentages for categorical variables. For continuous variables, one-way ANOVA or Kruskal–Wallis tests were used for four-group comparisons, and for categorical variables, *χ*^2^ tests or Fisher’s exact tests were applied, as appropriate. Age-adjusted incidence rates per 100,000 men for more aggressive and metastatic prostate cancer were calculated by region and rurality using the direct method, standardized to the 2000 U.S. standard population. Age was grouped in five-year intervals, and age-specific rates were weighted by the corresponding age distribution in the standard population to ensure comparability across geographic groups and time. To evaluate temporal trends in age-adjusted incidence rates, we performed joinpoint regression analysis using the Joinpoint Regression Program (Version 5.3.0, National Cancer Institute; RRID: SCR_018129). Annual percentage changes (APCs) and their 95% confidence intervals (CI) were calculated for each time segment. Multivariable logistic regression analyses were conducted to assess geographical disparities, adjusting for potential risk factors. Specifically, we considered and fitted two models to assess the associations between geographic or racial factors and prostate cancer aggressiveness: Model 1 included age, race, ethnicity, and geographical region, while Model 2 incorporated additional covariates such as serum PSA at diagnosis, lymph node positive status, year of diagnosis, and the interaction between geographic region and race. Odds ratios with 95% confidence intervals were reported. Significance was determined using two-sided tests with a significance level of 0.05. Data analysis was performed using R version 4.1.8 (Research Resource Identifier [RRID]: SCR_001905). Geographical analysis was done using ArcGIS Pro 3.6.1 (RRID: SCR_011081).

## Results

Using 2004–2022 SEER data, we identified and included 951,965 men, aged≥40 years, with primary prostate cancer (Table 1). White men accounted for 77.4% of prostate cancer cases overall and constituted the majority in each geographic region. The proportion of White men was highest in rural Appalachia (95.0%), followed by rural non-Appalachia (84.1%), and the proportions were relatively lower in urban Appalachia (79.8%) and urban non-Appalachia (76.2%). The percentages of Black and Hispanic men diagnosed with prostate cancer varied by region, with the highest representation in urban non-Appalachia (15.1% and 10.8%, respectively) and the lowest in rural Appalachian areas (4.2% and 0.8%, respectively). Men in rural areas were more likely to be diagnosed with more aggressive prostate cancer, to be diagnosed at an older age, and less likely to receive definitive treatment compared to men in urban areas.

**Table 1:** Study Characteristics of 2004–2022 Prostate Cancer Cases Aged 40+ by Geographical Region of Residence at the Time of Diagnosis

	Overall (N=951,965)	Rural Appalachia (N=19,034)	Rural non-Appalachia (N=90,253)	Urban Appalachia (N=29,184)	Urban non-Appalachia (N=813,494)
Age at diagnosis (yrs)					
Mean (SD)	66.6 (8.92)	67.3 (8.58)	67.4 (8.72)	65.7 (8.82)	66.6 (8.95)
Median (25th-75th percentile)	66.0 [40, 90]	67.0 [40, 90]	67.0 [40, 90]	66.0 [40, 90]	66.0 [40, 90]
Race (N [%])					
White	736,737 (77.4%)	18,075 (95.0%)	75,903 (84.1%)	23,284(79.8%)	619,475 (76.2%)
Black	140,091 (14.7%)	808 (4.2%)	11,247 (12.5%)	5012 (17.2%)	123,024 (15.1%)
Other	54,299 (5.7%)	45 (0.2%)	2389 (2.6%)	669 (2.3%)	51,196 (6.3%)
Unknown	20,838 (2.2%)	106 (0.6%)	714 (0.8%)	219 (0.8%)	19,799 (2.4%)
Ethnicity (N [%])					
Hispanic	92,931 (9.8%)	153 (0.8%)	3508 (3.9%)	1155 (4.0%)	88,115 (10.8%)
Non-Hispanic	859,034 (90.2%)	18,881 (99.2%)	86,745 (96.1%)	28,029(96.0%)	725,379 (89.2%)
Marital status (N [%])					
Ever married or living as married	728,196 (76.5%)	15,562 (81.8%)	70,980 (78.6%)	23,128(79.2%)	618,526 (76.0%)
Single—never married	98,393 (10.3%)	1098 (5.8%)	8497 (9.4%)	2055 (7.0%)	86,743 (10.7%)
Unknown	125,376 (13.2%)	2374 (12.5%)	10,776 (11.9%)	4001 (13.7%)	108,225 (13.3%)
Serum PSA (ng/ml)					
Mean (SD)	12.8 (19.1)	13.9 (20.5)	14.5 (20.8)	11.6 (17.5)	12.7 (18.9)
Median (25th-75th percentile)	6.70 [0.1, 98]	6.80 [0.1, 98]	7.20 [0.1, 98]	6.30 [0.1, 98]	6.60 [0.100, 98]
Missing	365,275 (38.4%)	7435 (39.1%)	34,418 (38.1%)	12,920(44.3%)	310,502 (38.2%)
Serum PSA (ng/ml) (N [%])					
High: 20+	71,314 (7.5%)	1599 (8.4%)	8271 (9.2%)	1645 (5.6%)	59,799 (7.4%)
Intermediate: ≥ 10 and < 20	94,080 (9.9%)	1973 (10.4%)	10,036 (11.1%)	2314 (7.9%)	79,757 (9.8%)
Low: < 10	421,296 (44.3%)	8027 (42.2%)	37,528 (41.6%)	12,305(42.2%)	363,436 (44.7%)
Unknown/missing	365,275 (38.4%)	7435 (39.1%)	34,418 (38.1%)	12,920(44.3%)	310,502 (38.2%)
Gleason score (N [%])					
6	352,322 (37.0%)	7071 (37.1%)	31,183 (34.6%)	10,920(37.4%)	303,148 (37.3%)
7	410,565 (43.1%)	7915 (41.6%)	38,991 (43.2%)	13,187(45.2%)	350,472 (43.1%)
8–10	183,684 (19.3%)	3953 (20.8%)	19,549 (21.7%)	4944 (16.9%)	155,238 (19.1%)
Unknown	5394 (0.6%)	95 (0.5%)	530 (0.6%)	133 (0.5%)	4636 (0.6%)
Tumor stage (N [%])					
T1	376,152 (39.5%)	8479 (44.5%)	34,167 (37.9%)	12,057(41.3%)	321,449 (39.5%)
T2	448,038 (47.1%)	8491 (44.6%)	45,069 (49.9%)	14,028(48.1%)	380,450 (46.8%)
T3	76,908 (8.1%)	1342 (7.1%)	7398 (8.2%)	1896 (6.5%)	66,272 (8.1%)
T4	19,263 (2.0%)	354 (1.9%)	1668 (1.8%)	381 (1.3%)	16,860 (2.1%)
Unknown	31,604 (3.3%)	368 (1.9%)	1951 (2.2%)	822 (2.8%)	28,463 (3.5%)
Distant metastasis (N [%])					
Yes	42,865 (4.5%)	949 (5.0%)	4612 (5.1%)	1017 (3.5%)	36,287 (4.5%)
No	876,391 (92.1%)	17,593 (92.4%)	82,823 (91.8%)	27,544(94.4%)	748,431 (92.0%)
Unknown	32,709 (3.4%)	492 (2.6%)	2818 (3.1%)	623 (2.1%)	28,776 (3.5%)
Aggressiveness (N [%])					
Less aggressive	559,837 (58.8%)	11,189 (58.8%)	51,164 (56.7%)	18,081(62.0%)	479,403 (58.9%)
More aggressive	353,069 (37.1%)	7180 (37.7%)	35,955 (39.8%)	10,042(34.4%)	299,892 (36.9%)
Unknown	39,059 (4.1%)	665 (3.5%)	3134 (3.5%)	1061 (3.6%)	34,199 (4.2%)
Lymph node (LN) (N [%])					
LN Positive No	614,956 (64.6%)	12,523 (65.8%)	58,799 (65.1%)	17,637(60.4%)	525,997 (64.7%)
LN Positive Yes	18,917 (2.0%)	297 (1.6%)	1832 (2.0%)	443 (1.5%)	16,345 (2.0%)
Unknown	318,092 (33.4%)	6214 (32.6%)	29,622 (32.8%)	11,104(38.0%)	271,152 (33.3%)
Definitive treatment received (N [%])					
Yes	609,195 (64.0%)	12,114 (63.6%)	56,190 (62.3%)	19,707(67.5%)	521,184 (64.1%)
No	342,719 (36.0%)	6914 (36.3%)	34,056 (37.7%)	9474 (32.5%)	292,275 (35.9%)
Unknown	51 (0.0%)	6 (0.0%)	7 (0.0%)	3 (0.0%)	35 (0.0%)

*SD* standard deviation

Unknown values for each study characteristic were excluded from tests of association with geographical region of residence at the time of diagnosis. All study characteristics were significantly associated with geographical region

The age-adjusted incidence rates of prostate cancer were calculated (269.23 per 100,000 men for the period 2004 to 2022) with details presented in the supplementary materials (Table S1, Figure S1; joinpoint analysis results in Figure S2). All regions except urban Appalachia showed a notable decline in incidence rates, followed by a slight rebound, with variation in the timing and magnitude of these changes. Rural Appalachia showed a consistent decrease over time (APC for 2004–2022: − 1.7%; 95% CI: [− 2.98%, − 0.42%]). In contrast, the other regions exhibited increases around 2014–2022 followed by sharp declines, for instance, rural non-Appalachia (APC for 2004–2013: − 3.27%; 95% CI: [− 15.36%, 7.39%]; APC for 2013–2022: 1.94%; 95% CI: [− 6.25%, 16.66%]), and urban non-Appalachia (APC for 2009–2014: − 8.54%; 95% CI: [− 13.29%, − 6.02%]; APC for 2014–2022: 2.45%; 95% CI: [1.10%, 4.19%]).

**Fig. 2:**
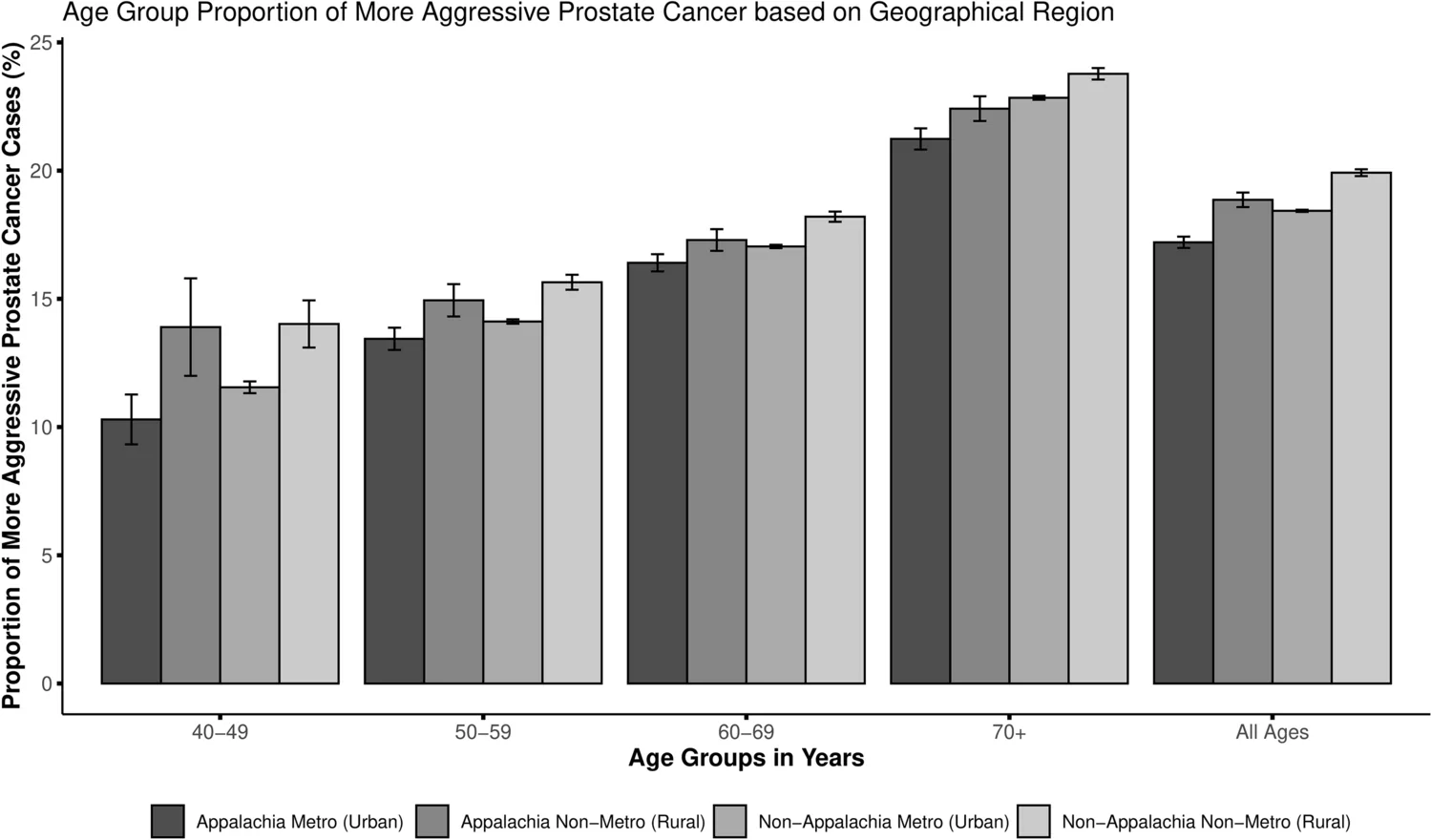
The proportion of more aggressive prostate cancer by age group and geographic region from 2004–2022

Regarding aggressive prostate cancer, we first examined the proportion of aggressive cases across age groups (40–49, 50–59, 60–69, and ≥70 years) among men diagnosed from 2004 to 2022. Men aged 70 years and above were more likely to be diagnosed with more aggressive prostate cancer (shown in Fig. 2 with detailed results presented in Table S2) compared to other age groups. When comparing geographic regions, those residing in rural non-Appalachia were more likely to be diagnosed with aggressive cancer compared to those in urban or rural Appalachia across years, with urban Appalachia showing the lowest proportion of aggressive cases. Also, among men aged 70+, those living in urban non-Appalachia had a higher proportion of aggressive cases at diagnosis than men in rural or urban Appalachia.

**Fig. 3:**
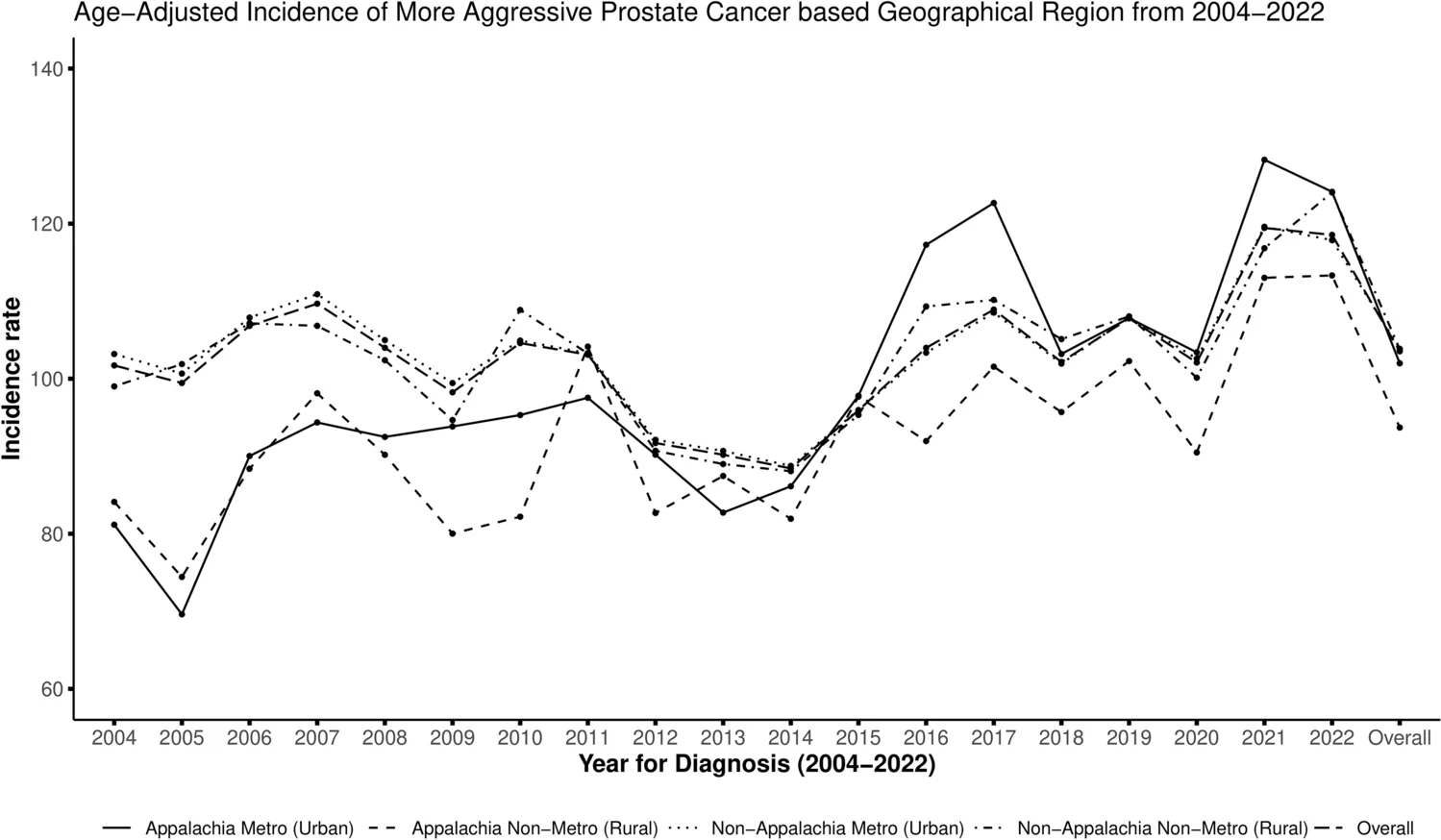
Age-Adjusted Incidence of More Aggressive Prostate Cancer by Geographical Region from 2004–2022

In addition, the age-adjusted incidence rate of more aggressive prostate cancer from 2004 to 2022 was 103.54 per 100,000 men, as shown in Fig. 3, with detailed data in Table S3, histograms in Figure S3, and joinpoint analysis results in Figure S4. Both urban and rural Appalachia had overall lower incidence rates of more aggressive prostate cancer (101.99 and 93.70 per 100,000 men, respectively) compared to urban and rural non-Appalachia (103.86 and 103.80 per 100,000 men, respectively). Of note, Appalachian regions experienced a significant increase in incidence over time, urban Appalachia (APC for 2004–2022: 2.73%; 95% CI: [1.20%, 4.65%]) and rural Appalachia (APC for 2004–2022: 1.91%; 95% CI: [0.61%, 3.39%]). We also observed increasing incidence rates in rural non-Appalachia over time (APC for 2004–2022: 0.97%; 95% CI: [0.11%, 1.90%]), although the increase was much slower than that in the Appalachian regions. Across the study period, differences between Appalachia and non-Appalachia narrowed, particularly during 2011–2015. However, after 2016, urban Appalachia showed a larger rise than the other regions, with notable increases in 2016, 2017, and 2021.

**Fig. 4:**
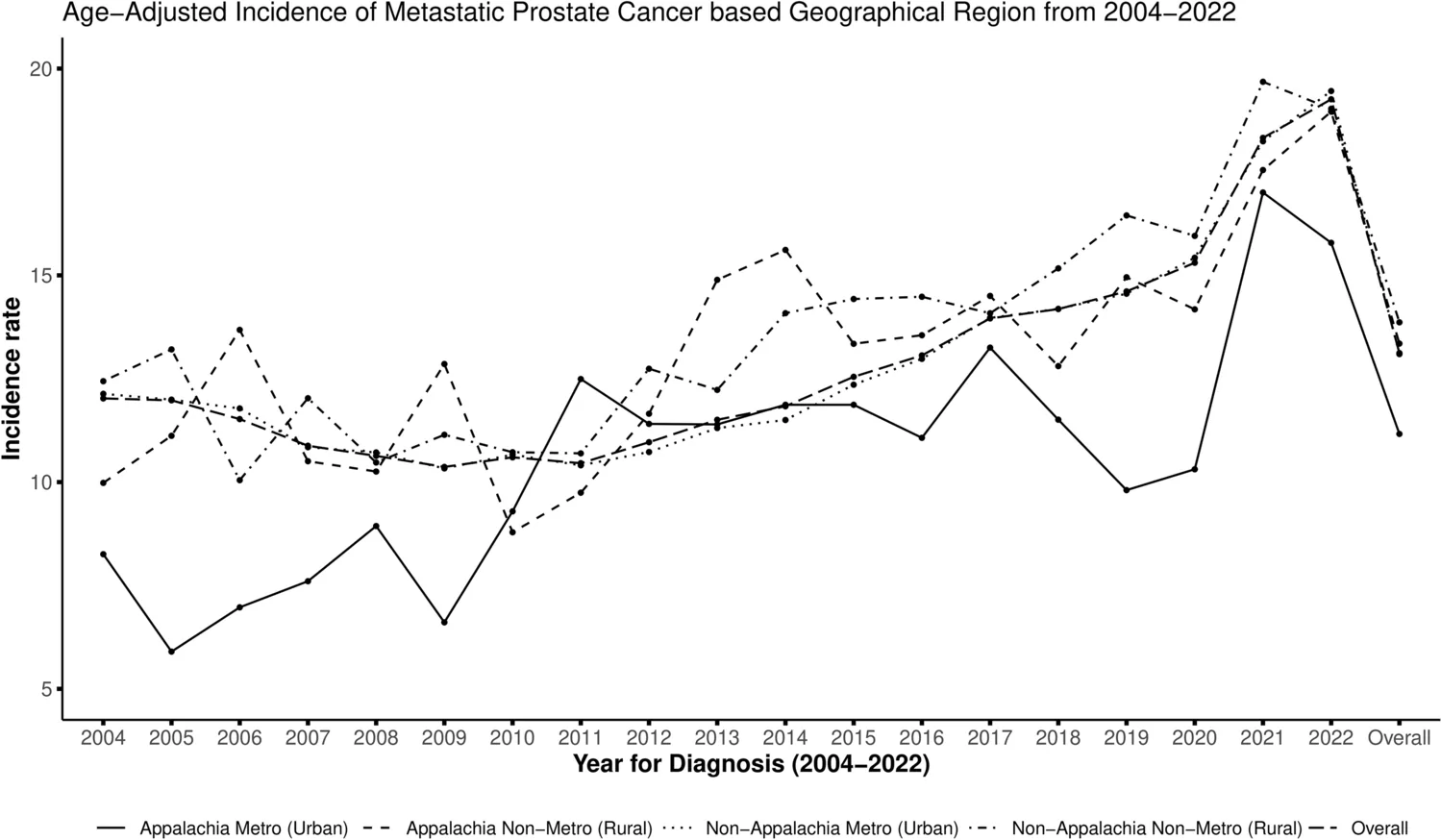
Age-Adjusted Incidence of Metastatic Prostate Cancer by Geographical Region from 2004–2022

Further, the overall age-adjusted incidence rate of metastatic prostate cancer from 2004 to 2022 was 13.12 per 100,000 men (shown in Fig. 4 with detailed results presented in Table S4 and histograms in Figure S5). Urban Appalachia and urban non-Appalachia (11.16 and 13.10 per 100,000 men, respectively) had lower incidence rates of metastatic prostate cancer compared to rural Appalachia and rural non-Appalachia (13.35 and 13.86 per 100,000 men, respectively). Generally, incidence rates declined through the late 2000 s and then increased, with notable regional variation: urban Appalachia showed the lowest rates during 2004–2009 but rose sharply by 2011 (from 6.61 per 100,000 men in 2009 to 12.49 per 100,000 men in 2011), while rural Appalachia increased in 2013–2014 (14.89–15.61 per 100,000 men) before a slight decline in 2015 (13.34 per 100,000 men). Importantly, all regions increased substantially after 2020, with the overall rate rising from 15.30 per 100,000 men (2020) to 18.32 per 100,000 men (2021) and 19.26 per 100,000 men (2022), which may reflect delayed diagnosis related to disruptions in PSA screening and care during the COVID-19 pandemic. This finding is also noted by the CDC and others that pandemic-related disruptions resulted in lower prostate cancer rates in 2020, with rates returning to or above pre-pandemic levels afterward [31, 32].

Multivariable logistic regressions were conducted to examine factors associated with prostate cancer aggressiveness (Table 2). From Model 1, men in rural non-Appalachia (OR = 1.118; 95% CI: [1.102, 1.135]) and rural Appalachia (OR = 1.105; 95% CI: [1.027, 1.092]) were more likely to be diagnosed with more aggressive prostate cancer compared to those in urban non-Appalachia, after adjusting for confounders. In contrast, men in urban Appalachia (OR = 0.937; 95% CI: [0.914, 0.961]) were less likely for diagnosis of more aggressive prostate cancer. Black men were more likely to have more aggressive cancer compared to White men, after adjusting for confounders in Model 1 (OR = 1.231; 95% CI: [1.216, 1.246]). In Model 2, the geographic associations among White men (reference group for race) were obtained directly from the main effects of geographic region. The results were similar to those in Model 1, with rural non-Appalachia having an OR of 1.077 (95% CI: 1.059, 1.096), rural Appalachia an OR of 1.035 (95% CI: 1.001, 1.069) and urban Appalachia an OR of 0.916 (95% CI: 0.889, 0.943) compared to urban non-Appalachia. Furthermore, for Black men, the geographic associations were estimated by considering the corresponding main effects and race-by-geographic region interactions. Compared to urban non-Appalachia, rural non-Appalachia and Urban Appalachia were not significantly associated with more aggressive prostate cancer, whereas rural Appalachia was associated with a lower odds of more aggressive prostate cancer (OR = 0.793; 95% CI: [0.677–0.928]). In addition, when examining the odds of more aggressive prostate cancer in urban non-Appalachia (reference group for geographical region), we found a significantly higher odds among Black men compared to White men (OR = 1.085; 95% CI: [1.069, 1.100]). A similar racial pattern was observed in urban Appalachia (OR = 1.160; 95% CI: [1.084, 1.242]). In contrast, in rural Appalachia, Black men had a lower odds of having more aggressive prostate cancer than White men (OR = 0.831; 95% CI: [0.708, 0.976]). However, in rural non-Appalachia, we did not observe a statistically significant racial difference in the odds of more aggressive prostate cancer.

**Table 2:** Multivariable Logistic Regressions Modeling Probability of More Aggressive Prostate Cancer at Diagnosis

Variables	Model 1: OR (95% CI)	Model 2: OR (95% CI)
Age at diagnosis	1.045 (1.044,1.046)	1.039 (1.038, 1.040)
Serum PSA (ng/mL)		
Low: < 10		Reference
Intermediate: ≥ 10 and < 20		2.086 (2.055, 2.119)
High: 20+		6.138 (6.016, 6.262)
Race		
White	Reference	Reference
Black	1.231 (1.216, 1.246)	1.085 (1.069, 1.100)
Other	1.430 (1.404, 1.456)	1.316 (1.290, 1.343)
Ethnicity		
Non-Hispanic	Reference	Reference
Hispanic	1.212 (1.195, 1.230)	1.094 (1.077, 1.111)
Lymph node positive status		
No		Reference
Yes		25.215 (23.578, 26.967)
Geographical region		
Urban non-Appalachia	Reference	Reference
Rural non-Appalachia	1.118 (1.102, 1.135)	1.077 (1.059, 1.096)
Urban Appalachia	0.937 (0.914, 0.961)	0.916 (0.889, 0.943)
Rural Appalachia	1.059 (1.027, 1.092)	1.035 (1.001, 1.069)
Year diagnosed		
2004		Reference
2005		1.065 (1.032, 1.098)
2006		1.073 (1.041, 1.106)
2007		1.098 (1.066, 1.132)
2008		1.207 (1.172, 1.244)
2009		1.313 (1.273, 1.353)
2010		1.328 (1.289, 1.369)
2011		1.324 (1.286, 1.364)
2012		1.469 (1.424, 1.514)
2013		1.541 (1.494, 1.589)
2014		1.698 (1.646, 1.752)
2015		1.794 (1.741, 1.850)
2016		1.887 (1.830, 1.944)
2017		1.985 (1.927, 2.045)
2018		0.572 (0.548, 0.598)
2019		0.570 (0.546, 0.595)
2020		0.588 (0.563, 0.614)
2021		0.577 (0.553, 0.602)
2022		0.570 (0.546, 0.594)
Race * geographical region		
Black, rural non-Appalachia		0.932 (0.891, 0.976)
Other, rural non-Appalachia		1.192 (1.087, 1.308)
Black, urban Appalachia		1.070 (0.998, 1.147)
Other, urban Appalachia		0.837 (0.706, 0.993)
Black, rural Appalachia		0.766 (0.652, 0.901)
Other, rural Appalachia		0.631 (0.325, 1.224)

The total sample size N=574,733 with 201,159 more aggressive cases and 373,574 less aggressive cases. The cases with unknown or missing values for the aggressiveness outcome were excluded. Model 1 includes basic demographic and geographic factors only; Model 2 includes the factors in Model 1 and also clinical presentation variables available at diagnosis and the interaction term. The results for the unknown category are not shown

*OR* odds ratio, *CI* confidence interval

## Discussion

In this study, we investigated geographic and racial disparities in prostate cancer incidence and disease aggressiveness. For the total population, men in Appalachia had lower incidence of aggressive prostate cancer at diagnosis compared to men in non-Appalachia, regardless of rurality. When metastatic prostate cancer, a subset of aggressive prostate cancer, was examined, men in urban areas had lower incidence rates compared to men in rural areas, regardless of Appalachia status. Compared to urban non-Appalachia, men in rural non-Appalachia or rural Appalachia were associated with more aggressive prostate cancer at diagnosis; and, urban Appalachia was associated with less aggressive prostate cancer at diagnosis, after adjusting for confounding factors. Black men were more likely to have more aggressive prostate cancer at diagnosis compared to White men, after adjusting confounding factors. When the interaction between race and geographical regions was examined, Black men were less likely to have more aggressive prostate cancer at diagnosis compared to White men in rural Appalachia; there was no racial difference in association in rural non-Appalachia.

These racial and geographical differences in patterns may reflect differences in population composition, social determinants of health, and/or access to medical care. Based on previous studies, rural Appalachia have shown to have a higher burden of cancer compared to urban non-Appalachia [12, 33]. In the present study, both rural and urban Appalachia had lower incidence rates of aggressive prostate cancer at diagnosis which is consistent with our previous study among men with an initial diagnosis of aggressive prostate cancer in Pennsylvania from 2004–2014 using the Pennsylvania Cancer Registry[19]. Appalachia has been shown to report lower screening rates for cancers such as breast and cervical cancer compared to the rest of the U.S.[34], and lower screening rates for prostate cancer (serum PSA test or digital rectal examination) compared to non-Appalachia [35]. Lower prostate cancer screening rates may be one possible reason for the lower incidence rates of aggressive prostate cancer at initial diagnosis in Appalachia compared to non-Appalachia. In the present study, both rural Appalachia and non-Appalachia had a higher incidence of metastatic disease at diagnosis compared to the urban areas; these regions were associated with more aggressive prostate cancer at diagnosis compared to urban non-Appalachia, after adjusting for demographic and clinical factors. These data suggest that men in rural areas may be diagnosed at even more advanced stages such as metastatic disease at initial diagnosis compared to men in urban areas including urban Appalachia. Possible reasons for the higher incidence of metastatic disease and positive association with aggressive disease at initial diagnosis in rural areas could be due to the lack of a timely diagnostic evaluation leading to more advanced stages of disease, specialist referral, and follow-up care because of longer travel distances as well as transportation limitations, fewer healthcare providers and specialties, and socioeconomic disadvantages[9, 10, 36–38].

Appalachian Regional Commission data indicate substantially lower availability of primary and specialty physicians in rural than in large metropolitan Appalachian counties (could you add the reference). Although urban Appalachian communities generally have greater proximity to healthcare resources, disparities related to insurance, neighborhood conditions, continuity of care, healthcare trust, and structural inequities may still contribute to the observed patterns [8, 12, 15, 39–42]. Also, rural Appalachia and rural non-Appalachia may represent distinct rural environments with differences in socioeconomic conditions, healthcare resources, and proximity to metropolitan areas. Grouping RUCC categories 4–9 into a single rural category may therefore mask important variation in rurality and healthcare access. Because our SEER analysis did not include detailed county-level socioeconomic, provider, travel, or screening measures, these potential mechanisms require further investigation and are the focus of ongoing work. In addition, Black men are known to have a higher incidence of prostate cancer and more aggressive disease compared to White men [43, 44]. In the present study, when the interaction between race and geographical region was considered, Black men were associated with more aggressive prostate cancer at initial diagnosis compared to White men in urban region (non-Appalachia and Appalachia), after adjusting confounding factors. However, Black men in rural Appalachia were less likely to have aggressive prostate cancer at initial diagnosis compared to White men in the same geographical region. Possible reasons for the lower odds of aggressive prostate cancer in Black men could be due to less prostate cancer screening in the rural areas as well as a smaller population in these regions compared to White men. Notably, Black men comprised of 12.5% in rural non-Appalachia and 4.2% rural Appalachia of this study population.

An important contextual factor during 2004–2022 is the evolution of prostate cancer screening guidelines. In 2008, the U.S. Preventive Services Task Force (USPSTF) recommended against routine PSA screening for men aged 75+, followed by a broader recommendation in 2012 against PSA-based screening for all men. These guideline shifts may reduce screening rates, particularly among older and high-risk populations, and may have contributed to delayed diagnosis and greater detection of aggressive disease at presentation. Some of the observed trends especially among rural and underserved populations, could partly reflect the downstream effects of these policy changes. Prior studies also indicate that rural residents face barriers to timely diagnosis and treatment, lower screening and follow-up rates, and shortages of urologists, all of which may contribute to more advanced disease at diagnosis and poorer outcomes [12, 15, 36, 37, 45–47]. From 2011 to 2015, the gap in prostate cancer incidence narrowed between Appalachia and non-Appalachia, following the enactment of Affordable Care Act (ACA) that increased funding for local health departments in Appalachia and expanded Medicaid eligibility, potentially improving the access to healthcare in underserved areas. However, Medicaid expansion was not uniform across SEER states; thus, other ACA-related mechanisms may also have contributed [38]. Further investigation is needed to delineate the specific mechanisms driving these regional trends.

There are several limitations of our study. The SEER program, covering approximately 26.5% of the U.S. population, does not capture the full geographic and demographic diversity of Appalachia, limiting generalizability, especially to more rural or socioeconomically disadvantaged areas not represented in SEER. Note that SEER database includes comprehensive state-wide data for two Appalachian states (Kentucky and New York) and also captures partial data from several other Appalachian states through regional registries (e.g., Georgia, North Carolina, Ohio, and Tennessee). Therefore, our results should be interpreted as applying to SEER-covered Appalachia. We also used the ARC 2008 county definition, despite later ARC updates, which may introduce some misclassification of Appalachian status. After 2014, Detroit, Michigan was excluded from SEER leading to a decreased sample size locations from older versions. In addition, rurality was defined using USDA 2013 RUCC (metro vs. non-metro), which may not capture temporal shifts in urbanization over 2004–2022. In 2023, RUCC defined using the urban area threshold, rose from 2,500 to 5,000 people, affecting the comparability with older RUCC versions. Moreover, unknown and missing SEER data, particularly for Gleason scores and tumor stage variables, may limit our analysis. However, only 4.1% of aggressiveness stages were unknown overall, and the proportion was similar across geographic groups (3.5%−4.2%), suggesting little differential missingness by geography, given the large analytic sample.

Despite these limitations, our findings provide evidence of meaningful racial and geographic disparities in aggressive prostate cancer at diagnosis. Although other studies such as Hurwitz et al. [48], have proposed narrower definitions of aggressiveness based on predicted mortality risk, our broader definition is consistent with prior work [19, 22, 30] and is based on SEER variables available at diagnosis, aiming to capture clinically meaningful aggressiveness and disparities in initial disease severity, rather than long-term outcomes. Also, given the large sample, relatively small associations may still reach statistical significance. Our findings are reported with consideration of both effect magnitude and precision. Although modest effect sizes may have limited clinical relevance, they may still be meaningful at the population level and may help identify geographic and racial disparities that warrant further investigation. Future work should evaluate alternative definitions of aggressiveness and assess the robustness of findings using updated Appalachian classifications and more refined rurality measures.

## Conclusions

In conclusion, using 2004–2022 SEER data, age-adjusted incidence analyses and multivariable logistic regression showed that prostate cancer burden and severity varied across geographic regions by Appalachia and rurality. Compared with urban non-Appalachia, rural regions, particularly rural non-Appalachia, had a higher odds of more aggressive disease, whereas urban Appalachia had a lower odds of more aggressive disease. In addition, the incidence of more aggressive prostate cancer increased over time in Appalachian areas, underscoring the need to monitor evolving temporal trends. Geographic disparities also varied by race: Black men generally had higher odds of more aggressive disease than White men in urban areas, while rural Appalachia showed a reversed association. Together, these findings support targeted, region- and race- specific strategies to improve early detection, screening access, and high-quality prostate cancer care.

## Supplementary Information

Below is the link to the electronic supplementary material.

Supplementary file1 (DOCX 260 KB)

## Data Availability

The data supporting the reported results are available from the Surveillance, Epidemiology, and End Results (SEER) database. The SEER is a publicly accessible database maintained by the National Cancer Institute (NCI), providing de-identified data for research purposes. The dataset used in this study can be accessed via the SEER website at https://seer.cancer.gov/, following the NCI’s data access and usage guidelines.
