# Supplementary material for: Geographic disparities and temporal trends in aggressive prostate cancer: a SEER population-based analysis (2004–2022)
Source: Cancer Causes Control. 2026 Oct 17;37(11):181. doi: 10.1007/s10552-026-02267-5 (PMC13676493; doi:10.1007/s10552-026-02267-5)
Supplement: Supplementary file 1 [file 10552_2026_2267_MOESM1_ESM.docx]

**Supplementary Materials for the paper entitled “Geographic Disparities and Temporal Trends in Aggressive Prostate Cancer: A SEER Population-Based Analysis (2004–2022)”**

Yifei Xu^1^, Ayush Singh^2^, Eric Li^3^, Nicholas George Zaorsky^2^, Nathaniel Geyer^4^, Yifan Yang^1^, Yanxu Yang^5^, Alicia McDonald^4^, Lijun Zhang^1^, Ming Wang^1*^

^1^ Department of Population and Quantitative Health Sciences, Case Western Reserve

University, 10900 Euclid Avenue, Cleveland, OH 44106, USA

^2^ Department of Radiation Oncology, University Hospitals Seidman Cancer Center, Case Western Reserve, University School of Medicine, Cleveland, OH

^3^ The University of Texas at Austin, 2515 Speedway, Austin, TX, USA

^4^ Department of Public Health Sciences, Pennsylvania State University College of

Medicine, 500 University Drive, Hershey, PA 17033, USA

^5^ Department of Pediatrics, Emory University School of Medicine, 2015 Uppergate Drive, Atlanta, GA 30322, USA

Disclaimers: Alicia McDonald, PhD, MPH is a full-time employee of Merck, Inc.

^*^ Corresponding Author:

Ming Wang, PhD

Department of Population and Quantitative Health Sciences

Case Western Reserve University School of Medicine

10900 Euclid Avenue

Robbins Building E240-C

Cleveland, Ohio 44106

Phone: 216-368-4526

Email: [mxw827@case.edu](mailto:mxw827@case.edu)

**Table S1.** Age-Adjusted Incidence of Prostate Cancer by Geographical Region (CI: confidence interval)

| **Year** | **Appalachia**  **Metro (Urban) rate (95% CI)** | **No. Events** | **Appalachia**  **Non-Metro (Rural) rate (95% CI)** | **No. Events** | **Non-Appalachia**  **Metro (Urban) rate (95% CI)** | **No. Events** | **Non-Appalachia**  **Non-Metro (Rural)**  **rate (95% CI)** | **No. Events** | **Overall**  **rate (95% CI)** | **No. Events** |
| --- | --- | --- | --- | --- | --- | --- | --- | --- | --- | --- |
| 2004 | 303.38 (283.67, 323.10) | 1038 | 281.91 (262.99, 300.84) | 909 | 333.98 (330.66, 337.29) | 40192 | 291.09 (282.40, 299.78) | 4387 | 327.37 (324.35, 330.38) | 46526 |
| 2005 | 292.10 (273.44, 310.76) | 1058 | 266.77 (248.90, 284.64) | 910 | 316.35 (313.16, 319.54) | 39025 | 286.40 (277.87, 294.94) | 4422 | 311.43 (308.52, 314.34) | 45415 |
| 2006 | 354.82 (335.09, 374.55) | 1383 | 310.07 (290.88, 329.25) | 1071 | 346.12 (342.82, 349.42) | 43734 | 308.39 (299.67, 317.11) | 4918 | 341.44 (338.42, 344.45) | 51106 |
| 2007 | 339.54 (320.88, 358.20) | 1419 | 323.04 (303.85, 342.24) | 1160 | 360.33 (357.00, 363.65) | 47016 | 319.40 (310.62, 328.19) | 5221 | 354.63 (351.60, 357.65) | 54816 |
| 2008 | 329.75 (311.98, 347.52) | 1468 | 275.05 (257.55, 292.55) | 1016 | 329.87 (326.75, 333.00) | 44662 | 294.79 (286.48, 303.10) | 4985 | 324.85 (322.00, 327.70) | 52131 |
| 2009 | 302.36 (285.59, 319.14) | 1397 | 242.44 (226.27, 258.62) | 923 | 321.91 (318.87, 324.95) | 45284 | 282.95 (274.91, 290.99) | 4927 | 315.31 (312.55, 318.07) | 52531 |
| 2010 | 288.63 (272.81, 304.46) | 1419 | 233.02 (217.68, 248.35) | 939 | 307.34 (304.41, 310.27) | 44553 | 279.59 (271.66, 287.52) | 4968 | 302.34 (299.67, 305.00) | 51879 |
| 2011 | 279.98 (264.55, 295.40) | 1409 | 280.97 (263.99, 297.95) | 1121 | 303.73 (300.86, 306.60) | 45466 | 268.57 (260.87, 276.26) | 4871 | 298.83 (296.21, 301.45) | 52867 |
| 2012 | 250.58 (236.49, 264.67) | 1335 | 216.55 (201.99, 231.11) | 901 | 249.53 (246.98, 252.08) | 38803 | 223.39 (216.46, 230.32) | 4169 | 246.17 (243.84, 248.50) | 45208 |
| 2013 | 241.14 (227.68, 254.59) | 1347 | 215.31 (200.96, 229.66) | 914 | 234.94 (232.51, 237.38) | 37728 | 215.26 (208.53, 221.98) | 4102 | 232.69 (230.46, 234.92) | 44091 |
| 2014 | 216.68 (204.14, 229.22) | 1252 | 195.61 (182.04, 209.18) | 842 | 213.39 (211.10, 215.67) | 35360 | 204.43 (197.92, 210.94) | 3957 | 212.20 (210.10, 214.30) | 41411 |
| 2015 | 229.92 (217.24, 242.60) | 1378 | 209.36 (195.46, 223.25) | 923 | 221.19 (218.90, 223.49) | 37724 | 214.80 (208.20, 221.41) | 4255 | 220.51 (218.40, 222.62) | 44280 |
| 2016 | 263.17 (249.75, 276.59) | 1614 | 205.44 (191.83, 219.05) | 925 | 231.63 (229.31, 233.94) | 40539 | 232.09 (225.27, 238.92) | 4660 | 231.85 (229.71, 233.98) | 47738 |
| 2017 | 257.51 (244.63, 270.39) | 1649 | 220.47 (206.54, 234.40) | 1009 | 239.02 (236.71, 241.34) | 42983 | 230.07 (223.35, 236.80) | 4710 | 238.31 (236.18, 240.44) | 50351 |
| 2018 | 269.30 (256.52, 282.07) | 1807 | 224.77 (210.90, 238.63) | 1055 | 235.58 (233.31, 237.85) | 43188 | 237.80 (231.02, 244.58) | 4951 | 236.67 (234.57, 238.77) | 51001 |
| 2019 | 274.78 (262.08, 287.49) | 1904 | 235.69 (221.58, 249.80) | 1120 | 247.06 (244.76, 249.35) | 46400 | 243.86 (237.04, 250.68) | 5144 | 247.37 (245.25, 249.49) | 54568 |
| 2020 | 257.15 (244.95, 269.34) | 1827 | 191.92 (179.28, 204.56) | 934 | 227.28 (225.03, 229.52) | 41410 | 215.75 (209.28, 222.22) | 4511 | 226.34 (224.28, 228.40) | 48682 |
| 2021 | 310.04 (296.75, 323.32) | 2243 | 239.17 (225.10, 253.24) | 1173 | 267.40 (264.98, 269.81) | 49484 | 259.39 (252.34, 266.44) | 5490 | 267.40 (265.18, 269.62) | 58390 |
| 2022 | 299.96 (287.16, 312.75) | 2237 | 239.49 (225.53, 253.45) | 1189 | 265.63 (263.25, 268.01) | 49943 | 262.58 (255.53, 269.63) | 5605 | 265.94 (263.75, 268.13) | 58974 |
| Overall | 279.01 (275.68, 282.35) | 29184 | 239.44 (235.95, 242.94) | 19034 | 271.45 (270.85, 272.05) | 813494 | 254.29 (252.60, 255.97) | 90253 | 269.23 (268.68, 269.78) | 951965 |

**Figure S1.** Line Plot of Age-Adjusted Incidence of Prostate Cancer by Geographic Region (2004–2022)

The age-adjusted incidence rate of prostate cancer from 2004–2022 was overall 269.23 per 100,000 men (**Figure S1**). Urban Appalachia and non-Appalachia (279.01 and 271.45 per 100,000 men respectively) had overall higher incidence rates than both rural Appalachia and non-Appalachia (239.44 and 254.29 per 100,000 men respectively). Rural Appalachia, in particular, predominantly had the lowest incidence rates, except for 2006, 2007, 2011, and 2013, when rural non-Appalachia had lower incidence rates.

**Figure S2.** Joinpoint Analysis of Age-Adjusted Incidence of Prostate Cancer by Geographic Region (2004–2022)

**Table S2.** The Proportion of More Aggressive Prostate Cancer by Age Group and Geographic Region (CI: confidence interval)

| 2004–2022 | **Appalachia Metro (Urban)** | | | **Appalachia Non-Metro (Rural)** | | | **Non-Appalachia Metro (Urban)** | | | **Non-Appalachia Non-Metro (Rural)** | | |
| --- | --- | --- | --- | --- | --- | --- | --- | --- | --- | --- | --- | --- |
| **Age at Diagnosis** | **No. of More Aggressive Cases for the Given Age**  **(Numerator)** | **No. of Cases for the Given Age**  **(Denominator)** | **%**  **(95% CI)** | **No. of More Aggressive Cases for the Given Age**  **(Numerator)** | **No. of Cases for the Given Age**  **(Denominator)** | **%**  **(95% CI)** | **No. of More Aggressive Cases for the Given Age**  **(Numerator)** | **No. of Cases for the Given Age**  **(Denominator)** | **%**  **(95% CI)** | **No. of More Aggressive Cases for the Given Age**  **(Numerator)** | **No. of Cases for the Given Age**  **(Denominator)** | **%**  **(95% CI)** |
| 40-49 | 201 | 976 | 10.30 (8.39, 12.20) | 92 | 331 | 13.90 (10.17, 17.62) | 4502 | 19489 | 11.55 (11.10, 12.00) | 399 | 1423 | 14.02 (12.22, 15.82) |
| 50-59 | 1655 | 6157 | 13.44 (12.59, 14.29) | 953 | 3189 | 14.94 (13.70, 16.18) | 45373 | 160726 | 14.12 (13.94, 14.29) | 4803 | 15349 | 15.65 (15.07, 16.22) |
| 60-69 | 4004 | 12204 | 16.40 (15.75, 17.06) | 2769 | 8006 | 17.29 (16.46, 18.12) | 115416 | 338611 | 17.04 (16.92, 17.17) | 13681 | 37578 | 18.20 (17.81, 18.59) |
| 70+ | 4182 | 9847 | 21.23 (20.43, 22.04) | 3366 | 7508 | 22.42 (21.47, 23.36) | 134601 | 294668 | 22.84 (22.69, 22.99) | 17072 | 35903 | 23.78 (23.33, 24.22) |
| All Ages | 10042 | 29184 | 17.20 (16.77, 17.64) | 7180 | 19034 | 18.86 (18.31, 19.42) | 299892 | 813494 | 18.43 (18.35, 18.52) | 35955 | 90253 | 19.92 (19.66, 20.18) |

**Table S3.** Age-Adjusted Incidence of More Aggressive Prostate Cancer by Geographical Region (CI: confidence interval)

| **Year** | **Appalachia**  **Metro (Urban) rate (95% CI)** | **No. Events** | **Appalachia**  **Non-Metro (Rural)**  **rate (95% CI)** | **No. Events** | **Non-Appalachia**  **Metro (Urban)**  **rate (95% CI)** | **No. Events** | **Non-Appalachia**  **Non-Metro (Rural)**  **rate (95% CI)** | **No. Events** | **Overall**  **rate (95% CI)** | **No. Events** |
| --- | --- | --- | --- | --- | --- | --- | --- | --- | --- | --- |
| 2004 | 81.17 (70.26, 92.07) | 247 | 84.11 (73.43, 94.80) | 258 | 103.19 (101.31, 105.06) | 12014 | 99.01 (93.89, 104.14) | 1465 | 101.71 (100.00, 103.43) | 13984 |
| 2005 | 69.61 (60.16, 79.07) | 239 | 74.43 (64.71, 84.16) | 243 | 100.67 (98.84, 102.49) | 12030 | 101.89 (96.74, 107.04) | 1538 | 99.45 (97.77, 101.12) | 14050 |
| 2006 | 90.03 (79.53, 100.53) | 321 | 88.39 (77.78, 99.00) | 290 | 107.88 (106.01, 109.76) | 13187 | 107.15 (101.93, 112.36) | 1668 | 106.82 (105.11, 108.53) | 15466 |
| 2007 | 94.36 (83.95, 104.76) | 361 | 98.12 (87.15, 109.09) | 334 | 110.90 (109.02, 112.78) | 13940 | 106.82 (101.67, 111.97) | 1702 | 109.68 (107.96, 111.39) | 16337 |
| 2008 | 92.49 (82.70, 102.29) | 386 | 90.19 (79.87, 100.51) | 318 | 104.98 (103.19, 106.77) | 13740 | 102.38 (97.40, 107.36) | 1682 | 103.99 (102.35, 105.63) | 16126 |
| 2009 | 93.84 (84.11, 103.57) | 409 | 80.02 (70.61, 89.43) | 299 | 99.44 (97.72, 101.16) | 13509 | 94.67 (89.94, 99.40) | 1598 | 98.25 (96.68, 99.82) | 15815 |
| 2010 | 95.31 (85.81, 104.82) | 441 | 82.20 (72.85, 91.55) | 317 | 104.92 (103.17, 106.67) | 14592 | 108.87 (103.85, 113.90) | 1878 | 104.60 (102.99, 106.20) | 17228 |
| 2011 | 97.54 (87.91, 107.18) | 446 | 104.14 (93.56, 114.72) | 402 | 103.30 (101.59, 105.01) | 14855 | 103.26 (98.41, 108.12) | 1814 | 103.10 (101.53, 104.67) | 17517 |
| 2012 | 90.20 (81.40, 99.00) | 451 | 82.68 (73.47, 91.90) | 331 | 92.12 (90.54, 93.71) | 13729 | 90.70 (86.22, 95.18) | 1645 | 91.71 (90.25, 93.16) | 16156 |
| 2013 | 82.73 (74.58, 90.88) | 439 | 87.45 (78.08, 96.83) | 358 | 90.69 (89.14, 92.23) | 13993 | 88.99 (84.60, 93.38) | 1649 | 90.19 (88.78, 91.61) | 16439 |
| 2014 | 86.13 (77.92, 94.33) | 472 | 81.94 (72.98, 90.90) | 342 | 88.75 (87.25, 90.26) | 14145 | 88.07 (83.72, 92.42) | 1646 | 88.43 (87.04, 89.81) | 16605 |
| 2015 | 97.80 (89.25, 106.36) | 559 | 97.69 (87.99, 107.38) | 416 | 95.97 (94.42, 97.51) | 15749 | 95.33 (90.88, 99.78) | 1846 | 95.93 (94.51, 97.34) | 18570 |
| 2016 | 117.27 (108.05, 126.48) | 691 | 91.95 (82.70, 101.21) | 404 | 103.36 (101.78, 104.94) | 17429 | 109.31 (104.58, 114.04) | 2156 | 103.98 (102.52, 105.44) | 20680 |
| 2017 | 122.67 (113.54, 131.80) | 752 | 101.55 (91.90, 111.20) | 448 | 108.53 (106.94, 110.12) | 18857 | 110.16 (105.47, 114.86) | 2218 | 108.92 (107.45, 110.38) | 22275 |
| 2018 | 103.19 (95.04, 111.35) | 660 | 95.70 (86.44, 104.95) | 432 | 101.95 (100.42, 103.48) | 17972 | 105.11 (100.57, 109.65) | 2150 | 102.16 (100.75, 103.57) | 21214 |
| 2019 | 107.82 (99.59, 116.05) | 709 | 102.28 (92.75, 111.80) | 467 | 107.93 (106.39, 109.48) | 19573 | 108.04 (103.47, 112.61) | 2235 | 107.79 (106.36, 109.21) | 22984 |
| 2020 | 103.40 (95.39, 111.41) | 695 | 90.49 (81.65, 99.32) | 429 | 102.59 (101.05, 104.14) | 17898 | 100.13 (95.67, 104.59) | 2040 | 102.09 (100.67, 103.50) | 21062 |
| 2021 | 128.24 (119.39, 137.09) | 876 | 113.01 (103.17, 122.86) | 538 | 119.60 (117.95, 121.25) | 21306 | 116.83 (112.06, 121.61) | 2427 | 119.45 (117.93, 120.96) | 25147 |
| 2022 | 124.11 (115.64, 132.59) | 888 | 113.32 (103.65, 123.00) | 554 | 117.87 (116.25, 119.49) | 21374 | 124.02 (119.13, 128.90) | 2598 | 118.55 (117.06, 120.04) | 25414 |
| Overall | 101.99 (99.90, 104.09) | 10042 | 93.70 (91.47, 95.94) | 7180 | 103.86 (103.48, 104.24) | 299892 | 103.80 (102.71, 104.89) | 35955 | 103.54 (103.19, 103.88) | 353069 |

**Figure S3.** Age-Adjusted Incidence of More Aggressive Prostate Cancer by Geographical Region (2004 – 2022)

**Figure S4.** Joinpoint Analysis of Age-Adjusted Incidence of More Aggressive Prostate Cancer by Geographical Region (2004–2022)

**Table S4.** Age-Adjusted Incidence of Metastatic Prostate Cancer by Geographical Region (CI: confidence interval)

| **Year** | **Appalachia**  **Metro (Urban) rate (95% CI)** | **No. Events** | **Appalachia**  **Non-Metro (Rural)**  **rate (95% CI)** | **No. Events** | **Non-Appalachia**  **Metro (Urban)**  **rate (95% CI)** | **No. Events** | **Non-Appalachia**  **Non-Metro (Rural)**  **rate (95% CI)** | **No. Events** | **Overall**  **rate (95% CI)** | **No. Events** |
| --- | --- | --- | --- | --- | --- | --- | --- | --- | --- | --- |
| 2004 | 8.26 (4.84, 11.67) | 25 | 9.98 (6.08, 13.89) | 28 | 12.13 (11.48, 12.79) | 1357 | 12.44 (10.61, 14.27) | 182 | 12.02 (11.42, 12.62) | 1592 |
| 2005 | 5.90 (3.10, 8.70) | 20 | 11.12 (7.20, 15.03) | 34 | 11.99 (11.35, 12.64) | 1387 | 13.21 (11.32, 15.10) | 192 | 11.98 (11.39, 12.57) | 1633 |
| 2006 | 6.97 (4.11, 9.83) | 25 | 13.68 (9.37, 18.00) | 42 | 11.78 (11.15, 12.41) | 1393 | 10.05 (8.40, 11.69) | 147 | 11.52 (10.95, 12.10) | 1607 |
| 2007 | 7.60 (4.47, 10.73) | 26 | 10.50 (6.79, 14.22) | 33 | 10.84 (10.25, 11.44) | 1323 | 12.03 (10.26, 13.79) | 183 | 10.88 (10.33, 11.43) | 1565 |
| 2008 | 8.94 (5.74, 12.14) | 34 | 10.26 (6.66, 13.85) | 35 | 10.72 (10.13, 11.30) | 1360 | 10.47 (8.86, 12.09) | 167 | 10.63 (10.10, 11.17) | 1596 |
| 2009 | 6.61 (3.95, 9.27) | 28 | 12.86 (8.85, 16.86) | 43 | 10.33 (9.77, 10.89) | 1356 | 11.14 (9.47, 12.82) | 177 | 10.37 (9.85, 10.89) | 1604 |
| 2010 | 9.29 (6.00, 12.58) | 36 | 8.79 (5.62, 11.95) | 33 | 10.66 (10.10, 11.23) | 1438 | 10.72 (9.12, 12.32) | 179 | 10.60 (10.08, 11.12) | 1686 |
| 2011 | 12.49 (8.83, 16.15) | 51 | 9.74 (6.38, 13.11) | 35 | 10.41 (9.85, 10.96) | 1448 | 10.69 (9.11, 12.28) | 183 | 10.46 (9.95, 10.97) | 1717 |
| 2012 | 11.41 (8.12, 14.69) | 53 | 11.65 (7.91, 15.39) | 41 | 10.73 (10.18, 11.28) | 1535 | 12.74 (11.03, 14.45) | 223 | 10.96 (10.45, 11.48) | 1852 |
| 2013 | 11.40 (8.29, 14.50) | 58 | 14.89 (10.85, 18.93) | 55 | 11.31 (10.75, 11.86) | 1669 | 12.23 (10.56, 13.90) | 215 | 11.51 (10.99, 12.03) | 1997 |
| 2014 | 11.87 (8.51, 15.23) | 54 | 15.61 (11.46, 19.77) | 58 | 11.50 (10.95, 12.05) | 1768 | 14.08 (12.30, 15.87) | 249 | 11.83 (11.32, 12.35) | 2129 |
| 2015 | 11.87 (8.71, 15.03) | 62 | 13.34 (9.48, 17.21) | 49 | 12.36 (11.79, 12.92) | 1913 | 14.43 (12.64, 16.21) | 263 | 12.55 (12.02, 13.07) | 2287 |
| 2016 | 11.07 (8.05, 14.09) | 58 | 13.55 (9.84, 17.27) | 54 | 12.98 (12.41, 13.55) | 2088 | 14.48 (12.71, 16.25) | 272 | 13.06 (12.53, 13.59) | 2472 |
| 2017 | 13.25 (10.13, 16.37) | 77 | 14.50 (10.76, 18.24) | 61 | 13.97 (13.38, 14.56) | 2308 | 14.08 (12.37, 15.79) | 274 | 13.96 (13.42, 14.50) | 2720 |
| 2018 | 11.51 (8.71, 14.31) | 70 | 12.80 (9.40, 16.21) | 57 | 14.19 (13.61, 14.77) | 2401 | 15.17 (13.40, 16.94) | 294 | 14.18 (13.65, 14.72) | 2822 |
| 2019 | 9.81 (7.30, 12.31) | 63 | 14.95 (11.17, 18.73) | 64 | 14.56 (13.98, 15.14) | 2536 | 16.45 (14.62, 18.27) | 326 | 14.61 (14.08, 15.15) | 2989 |
| 2020 | 10.31 (7.71, 12.91) | 66 | 14.18 (10.57, 17.78) | 64 | 15.43 (14.81, 16.04) | 2557 | 15.95 (14.14, 17.77) | 311 | 15.30 (14.74, 15.87) | 2998 |
| 2021 | 17.00 (13.59, 20.42) | 106 | 17.55 (13.47, 21.63) | 77 | 18.24 (17.58, 18.90) | 3089 | 19.68 (17.66, 21.70) | 387 | 18.32 (17.71, 18.94) | 3659 |
| 2022 | 15.79 (12.60, 18.98) | 105 | 18.96 (14.80, 23.12) | 86 | 19.46 (18.78, 20.13) | 3361 | 19.03 (17.08, 20.97) | 388 | 19.26 (18.64, 19.87) | 3940 |
| Overall | 11.16 (10.44, 11.89) | 1017 | 13.35 (12.47, 14.23) | 949 | 13.10 (12.96, 13.23) | 36287 | 13.86 (13.46, 14.27) | 4612 | 13.12 (12.99, 13.25) | 42865 |

**Figure S5.** Age-Adjusted Incidence of Metastatic Prostate Cancer by Geographical Region (2004-2022)
